# Supplementary material for: Compass-like manipulation of electronic nematicity in Sr3Ru2O7
Source: Proc Natl Acad Sci U S A. 2023 Aug 28;120(36):e2308972120. doi: 10.1073/pnas.2308972120 (PMC10483601; doi:10.1073/pnas.2308972120)
Supplement: Supplementary file 1 — Appendix 01 (PDF) [file pnas.2308972120.sapp.pdf]

Supplementary material for 'Compass-like manipulation of electronic nematicity in $Sr_3Ru_2O_7$'

Masahiro Naritsuka,[1,*] Izidor Benedičič,[1,*] Luke C. Rhodes,[1] Carolina A. Marques,[1] Christopher Trainer,[1] Zhiwei Li,[2,†] Alexander C. Komarek,[2] and Peter Wahl[1,‡]

[1]SUPA, School of Physics and Astronomy, University of St Andrews, North Haugh, St Andrews, Fife, KY16 9SS, United Kingdom

[2]Max Planck Institute for Chemical Physics of Solids, Nöthnitzer Straße 40, 01187 Dresden, Germany

[*]These authors contributed equally.

[†]Current address: Key Lab for Magnetism and Magnetic Materials of the Ministry of Education, Lanzhou University, Lanzhou 730000, China

[‡]Correspondence to: wahl@st-andrews.ac.uk

S1. CHARACTERIZATION OF SAMPLE

Single crystal samples of $Sr_3Ru_2O_7$ used for the experiments here were from the same batch as the ones used in Ref. [1]. Samples were characterized by resistivity (RRR $\sim$ 102) and specific heat measurements as well as Transmission Electron Microscopy. These results are available in section S1 of the supplementary material of Ref. 1.

A. Magnetization measurements

The in-plane field dependence of the metamagnetic transitions of $Sr_3Ru_2O_7$ were characterized by magnetization measurements. The measurements were carried out using a Quantum Design MPMS3 SQUID magnetometer capable of applying a magnetic field of up to 7T and equipped with a sample rotator. The sample was mounted on the rotator such that the field could be applied in any direction in the sample $a - b$ plane. The samples were cut along the edges of the tetragonal unit cell ([1 0 0] and [0 1 0] directions in the orthorhombic unit cell). The alignment was checked using X-ray diffraction. When mounting the sample for measurements, the edges were aligned with the straight edges of the sample stage of the rotator, thus allowing the field direction for the measurement to be determined relative to the crystallographic directions. To account for the diamagnetic background of the sample rotator, the measurements presented in the following were repeated with empty rotator only, allowing for subtraction of the background signal from the rotator.

The measurements in the MPMS were conducted at 1.8K. The sample was rotated through a 180° angle in 6.4° steps. For each 6.4° increment the field was ramped from 4.5T to 7T, the field range of the metamagnetic transitions in Sr_3RuO_7. The derivative of the magnetization data, i.e. the magnetic susceptibility of the sample, is plotted as a color plot in Fig. S1a. The magnetization data for fields applied along the different crystallographic high-symmetry directions is shown in Fig. S1b. The resulting susceptibility curves are shown in the inset of Fig. S1b. The two metamagnetic transitions appear as peaks in the

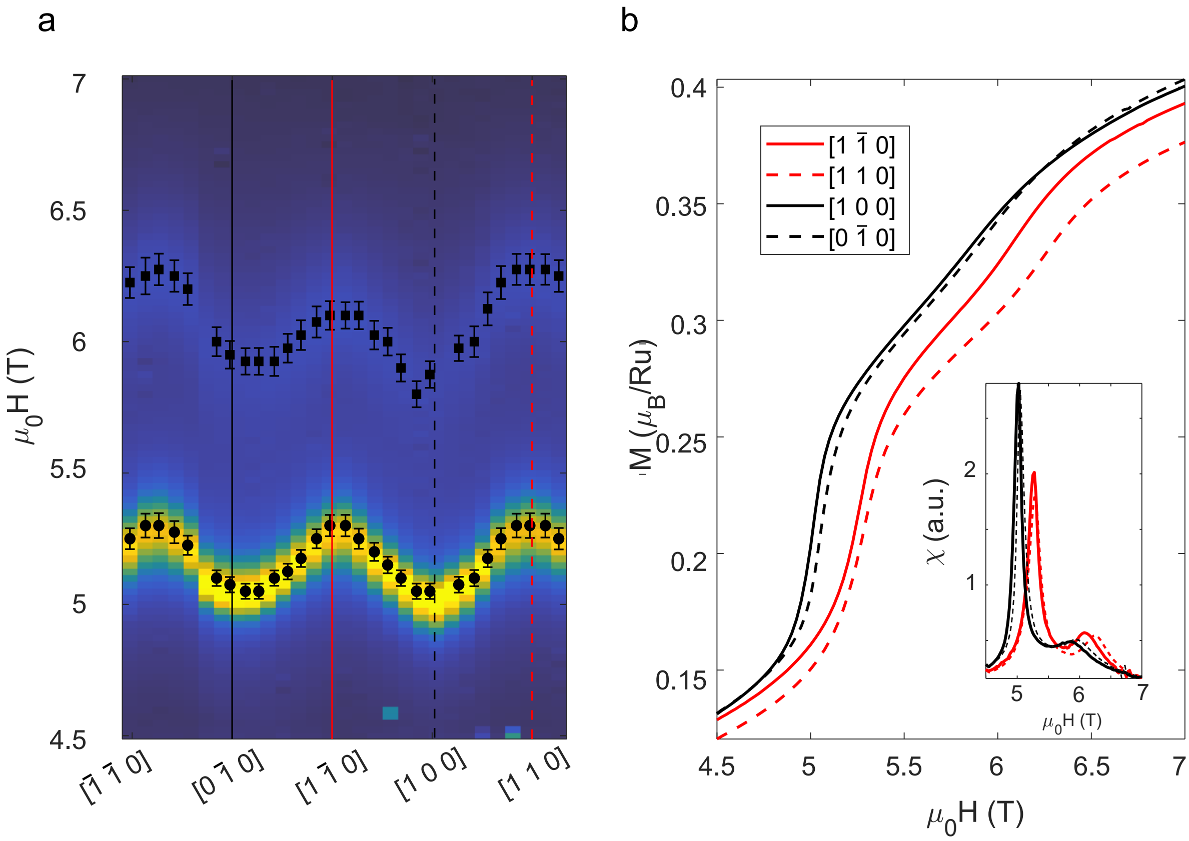

FIG. S1: **Angle dependence of in-plane magnetization.** (a) Color plot of the magnetic susceptibility of $Sr_3Ru_2O_7$ as a function of field angle in the sample $a - b$ plane, the fields at which the metamagnetic transitions occur are plotted as points on top of the color map plot. The metamagnetic transitions and their associated errors are determined by fitting a Gaussian function to the peaks in the magnetic susceptibility. (b) Plot of the magnetization for fields applied along the orthorhombic ([1 1 0], [1 $\bar{1}$ 0]) and tetragonal ([1 0 0], [0 $\bar{1}$ 0]) axes. The inset shows the corresponding magnetic susceptibility curves.

magnetic susceptibility, the first as a sharp peak at $\sim$ 5T and the second as a broader peak slightly above 6T. We determine the field at which the metamagnetic transition occurs by fitting a Gaussian function to these peaks.

From the results of the magnetization measurement, we find that the magnitude of the field required to induce the metamagnetic transition depends on the orientation of the

field in the crystallographic $a-b$ plane. The first transition at about 5T exhibits a four-fold symmetry with respect to the field direction. The transition occurs at higher fields for field along the orthorhombic $[1\,1\,0]$ and $[1\,\bar{1}\,0]$ directions, and at lower field along the tetragonal axes ($[1\,0\,0]$ and $[0\,1\,0]$). The transition at higher fields (around 6T) exhibits only a two-fold symmetry as a function of field angle. The maximum field required to induce this transition is for fields along the orthorhombic axes ($[1\,1\,0]$ and $[1\,\bar{1}\,0]$), but for one of them, the transition occurs for a lower field than for the other.

S2. DATA PROCESSING

A. Determination of nematic order parameter Ψ

The following assumptions are made in defining the nematic order parameter Ψ: 1) the shape of the scattering pattern around the defect is rectangular; 2) the directions of the edges of the rectangle are always along the crystallographic $a-$ and $b-$axis; 3) $a > b$ at zero field. Ψ is defined as,

$$\Psi = \arctan\frac{a}{b} - 45^\circ,$$

so that Ψ becomes zero when scattering from the defect becomes four-fold symmetric.

The dependence of the order parameter on the magnetic field strength when the magnetic field is applied in the orthorhombic directions ($[1\,1\,0]$ and $[1\,\bar{1}\,0]$) is shown in Fig. S2. The magnetic field strength at which the order parameter becomes zero is not strongly affected by the magnetic field strength in the c-axis direction.

B. Phase-referenced Fourier transformation

In Fig. 2j, we show that the intensity and relative phase of the checkerboard charge order varies periodically with the direction of the applied in-plane magnetic field. The surface layer of $Sr_3Ru_2O_7$ has two inequivalent but symmetry-related Sr sites due to the rotation of the RuO_6 octahedra around the c-axis. This is reflected in the checkerboard

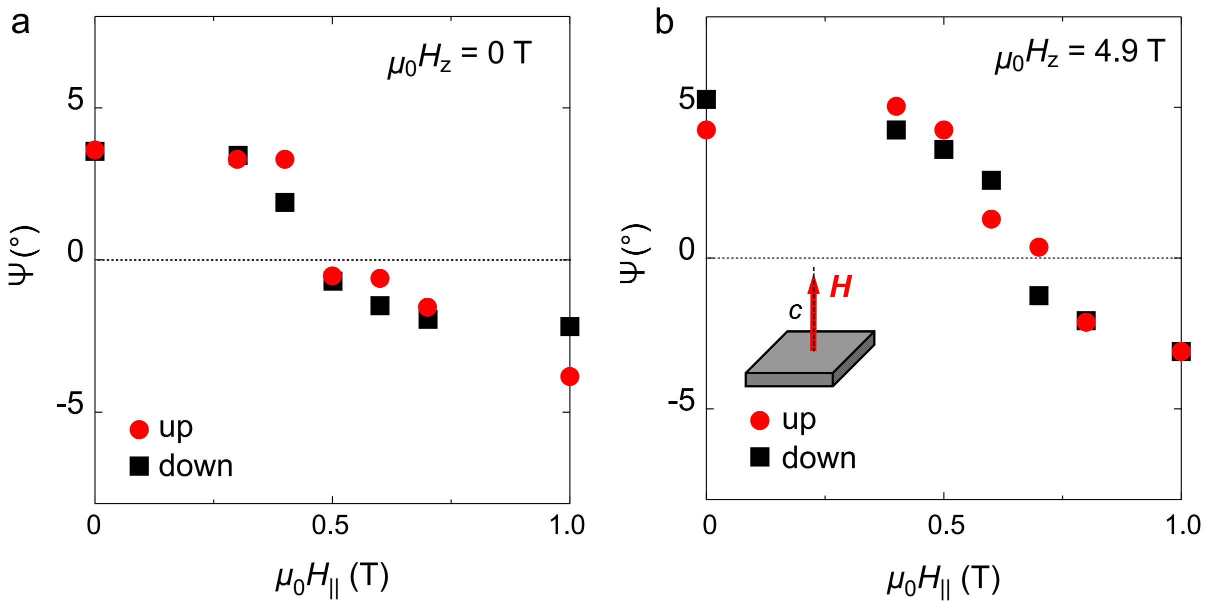

FIG. S2: **Nematic order parameter Ψ as a function of the in-plane field $\mu_0 H_{||}$.** (a, b) Order parameter Ψ as a function of in-plane field for an out-of-plane component of the field of (a) $\mu_0 H_z = 0$T and (b) $\mu_0 H_z = 4.9$T. The in-plane field is applied in the in-plane direction normal to the preferred direction of the nematicity in zero field ($\varphi = 45°$). The field at which the nematicity switches direction, i.e. $\Psi \rightarrow 0$, is ~ 0.5T for $\mu_0 H_z = 0$T (a) and ~ 0.6T for $\mu_0 H_z = 4.9$T (b). The upsweep (red circles) and downsweep (black rectangles) of the magnetic field are almost identical, showing, within the error of our measurements, no indication of hysteresis.

pattern of the brighter and darker Sr atoms in topographic images. When the field direction is set to $\varphi = 0°$, Sr atoms at A sites are brighter than ones at B sites. As φ increases, the difference in brightness between the two sites becomes smaller toward $\varphi = 45°$. With further rotation from $\varphi = 45°$ to $\varphi = 90°$, the B sites become brighter than the A sites. The reveral of the contrast between the two sublattices can be clearly seen from Fig. 2b, d.

In order to capture this behavior of the checkerboard charge order quantitatively, we use a phase-referenced Fourier transformation of topographic images. The checkerboard

charge order appears as two pairs of Bragg peaks at $\mathbf{q}_{\rm ckb} = (\pm 1/2, \pm 1/2)$. The intensity of the checkerboard appears in the intensity of the Fourier peak at $\mathbf{q}_{\rm ckb}$. The relative phase change is reflected in the information of the phase of the Fourier peaks. The phase is represented by θ in the following,

$$\theta(\mathbf{q}_{\rm ckb}, \varphi) = \arccos\left(\mathrm{Re}\left[\frac{\tilde{z}(\mathbf{q}_{\rm ckb}, \varphi)}{|\tilde{z}(\mathbf{q}_{\rm ckb}, \varphi)|}\right]\right),$$

where $\tilde{z}(\mathbf{q}, \varphi)$ is the Fourier transform of the topography obtained at field angle φ. The choice of the origin of the phase is arbitrary, so for simplicity, the phase θ is referenced relative to $\varphi = 0°$. As shown in Fig. 2j, $\cos(\theta(\mathbf{q}_{\rm ckb}, \varphi)) = -1$ at $\varphi = 0°$, $180°$ and $360°$, and $\cos(\theta(\mathbf{q}_{\rm ckb}, \varphi)) = 1$ at $\varphi = 90°$, $270°$. Four sign reversals are observed around $\varphi = 45°$, $135°$, $225°$, $315°$ while the magnetic field is rotated by $360°$.

To be able to compare the phase between consecutive topographic images, we apply the Lawler algorithm[2] to correct for any drift and cropped the exact same region around a set of defects from topographic images, ensuring that the global phase is the same across the whole data set.

C. Processing of QPI data

All spectroscopic maps acquired for the four different in-plane directions of the magnetic field shown in Fig. 4a-d were performed at the same location of the sample, using the same tip and the same measurement conditions (set point, temperature), changing only the magnetic field direction. The resulting maps were cropped to the exact same region, so that the Fourier transformations show quasi-particle interference originating from the same atomic-scale area of the sample. To correct for minor drift, the atomic peaks were mapped to be exactly on a square with same lattice constant. Finally, a rotation operation was performed so that the Sr-Sr direction is aligned with the vertical and horizontal directions of the image. No symmetrization was performed on any of the QPI data. The resulting dI/dV map is shown in Fig. S5. In the main text, we show $\mathrm{d}\ln I/\mathrm{d}\ln V(V) = \frac{(\mathrm{d}I/\mathrm{d}V)(V)}{I(V)/V}$ to remove the influence from the set-point effect.

S3. TIGHT-BINDING MODELS

A. Band structure plots with orbital character

In addition to band structure plots in Fig. 3 in the main text, we show in Fig. S3 band structures plotted on high-symmetry paths of a one-atom unit cell. The orbital characters are indicated by the colour of the bands.

To demonstrate that our minimal tight-binding model can reproduce the main features of the low-energy electronic structure, in Fig. S4 we calculate the cLDOS with magnetisation tilted out of the *ab*-plane and compare it with the case with magnetisation completely lying within the plane. With tilted magnetisation, we observe emergence of stripes connecting the Sr atoms in $[\bar{1}\,1\,0]$ direction. This is qualitatively consistent with previous experimental reports [1] where the emergence of stripe order with out-of-plane magnetic field was observed, providing further evidence for the validity of our model.

---

[1] Carolina A. Marques, Luke C. Rhodes, Izidor Benedičič, Masahiro Naritsuka, Aaron B. Naden, Zhiwei Li, Alexander C. Komarek, Andrew P. Mackenzie, and Peter Wahl. Atomic-scale imaging of emergent order at a magnetic field–induced Lifshitz transition. *Sci. Adv.*, 8:eabo7757, 2022. doi: 10.1126/sciadv.abo7757. URL `https://www.science.org/doi/10.1126/sciadv.abo7757`.

[2] M. J. Lawler, K. Fujita, Jhinhwan Lee, A. R. Schmidt, Y. Kohsaka, Chung Koo Kim, H. Eisaki, S. Uchida, J. C. Davis, J. P. Sethna, and Eun-Ah Kim. Intra-unit-cell electronic nematicity of the high-Tc copper-oxide pseudogap states. *Nature*, 466(7304):347–351, July 2010. ISSN 0028-0836, 1476-4687. doi: 10.1038/nature09169. URL `http://www.nature.com/doifinder/10.1038/nature09169`.

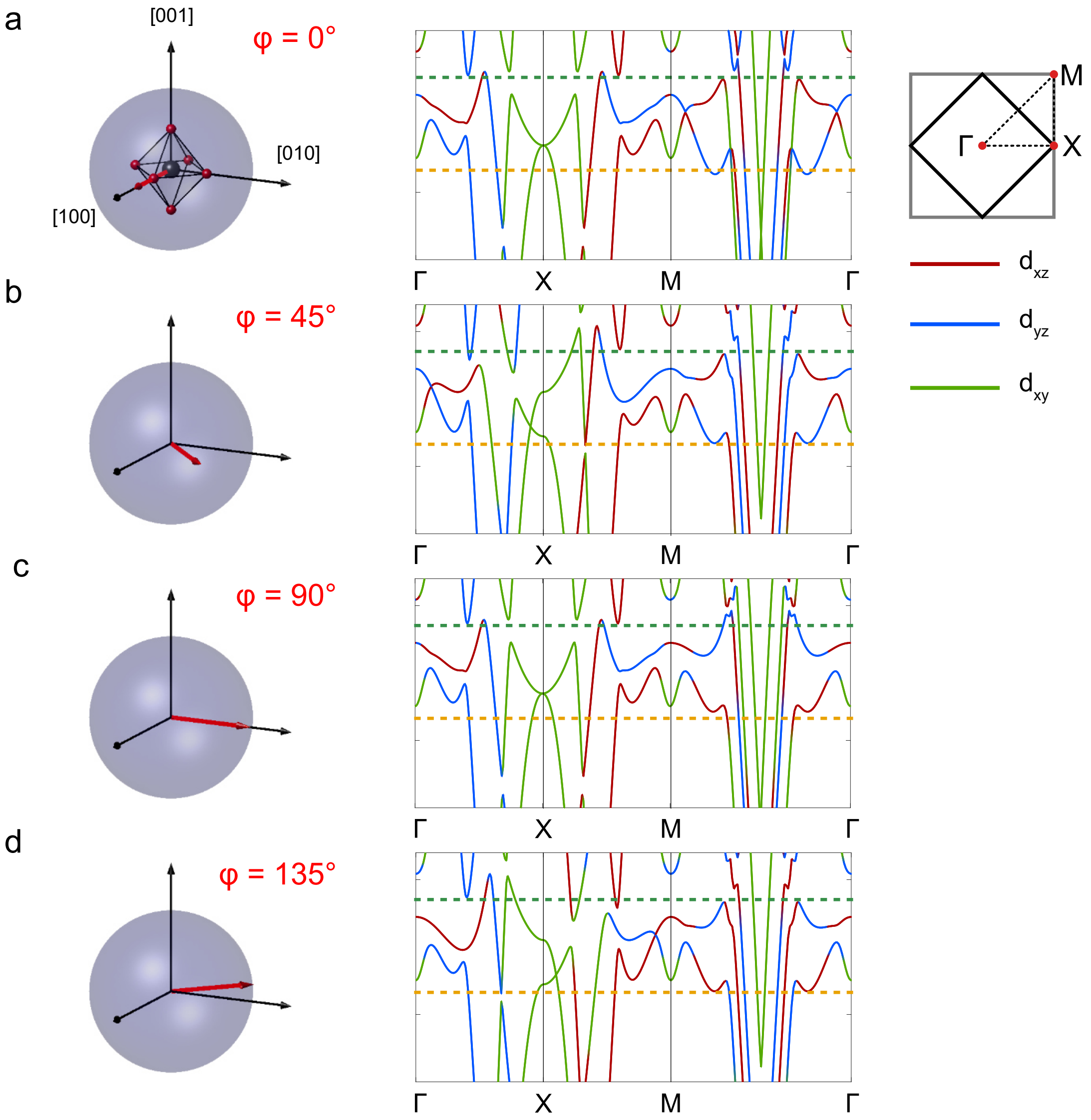

FIG. S3: **Orbitally-resolved band structure with different magnetisation directions a-d**, Band structure plotted in whole one-atom unit cell Brillouin zone. Bands color at each k-point is determined by its maximum orbital character.

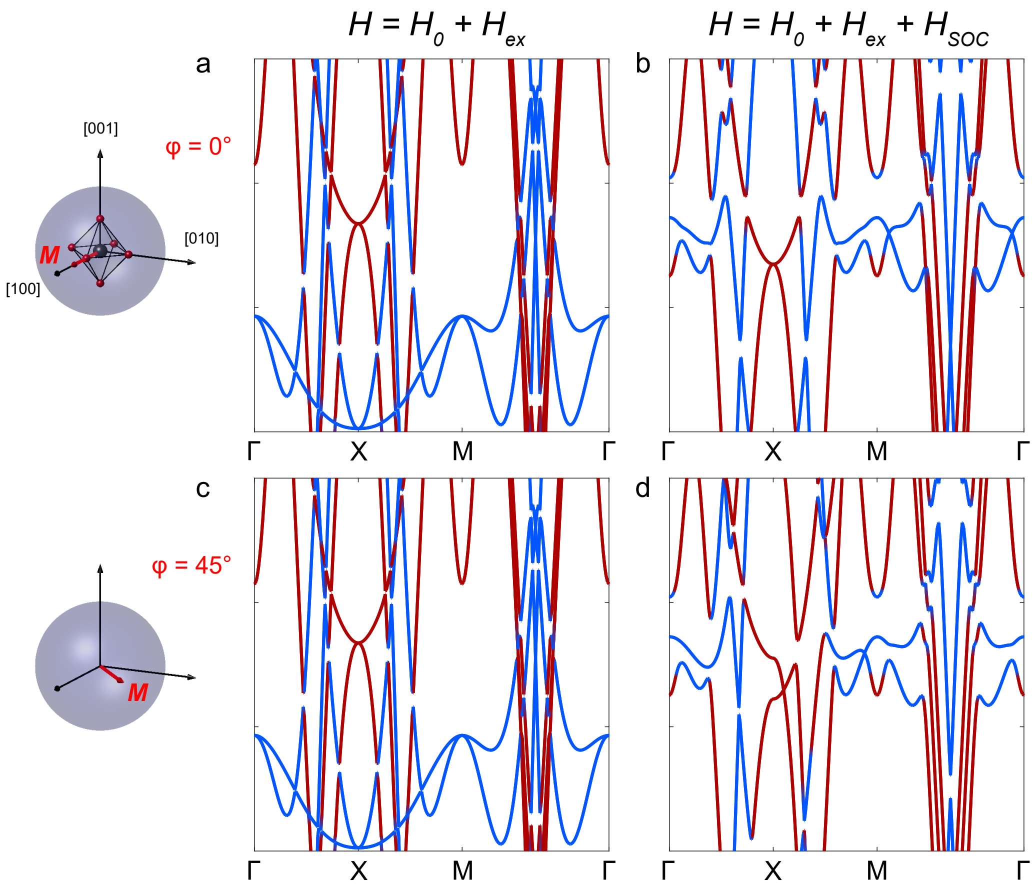

FIG. S4: **Effects of magnetisation and spin-orbit interaction a**, Band structure plotted in whole one-atom unit cell Brillouin zone, with $\vec{M} \parallel [1\,0\,0]$ but without spin-orbit coupling. Red and blue colours denote majority and minority spin character, respectively. **b**, Band structure with $\vec{M} \parallel [1\,0\,0]$ and spin-orbit interaction included in the calculation. **c**, Band structure with $\vec{M} \parallel [1\,1\,0]$ without spin-orbit coupling. **d**, Band structure with $\vec{M} \parallel [1\,1\,0]$ and spin-orbit interaction included in the calculation.

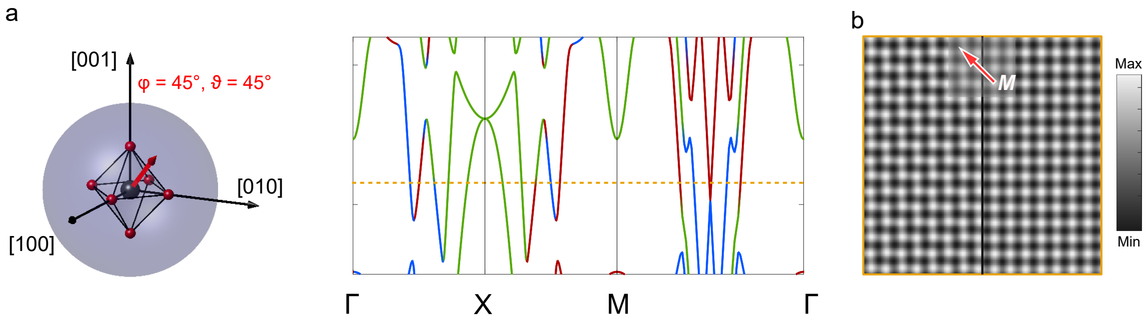

FIG. S5: **Electronic structure with out-of-plane tilting a**, Band structure of a minimal model with magnetisation tilted by 45° from the ab plane. **b**, cLDOS calculated at energy indicated by orange dashed line in **a**. Left: cLDOS of a model with magnetisation tilted by $\vartheta = 45°$ from the ab plane. Right: cLDOS of a model with magnetisation lying entirely within the ab plane, $\theta = 0°$.

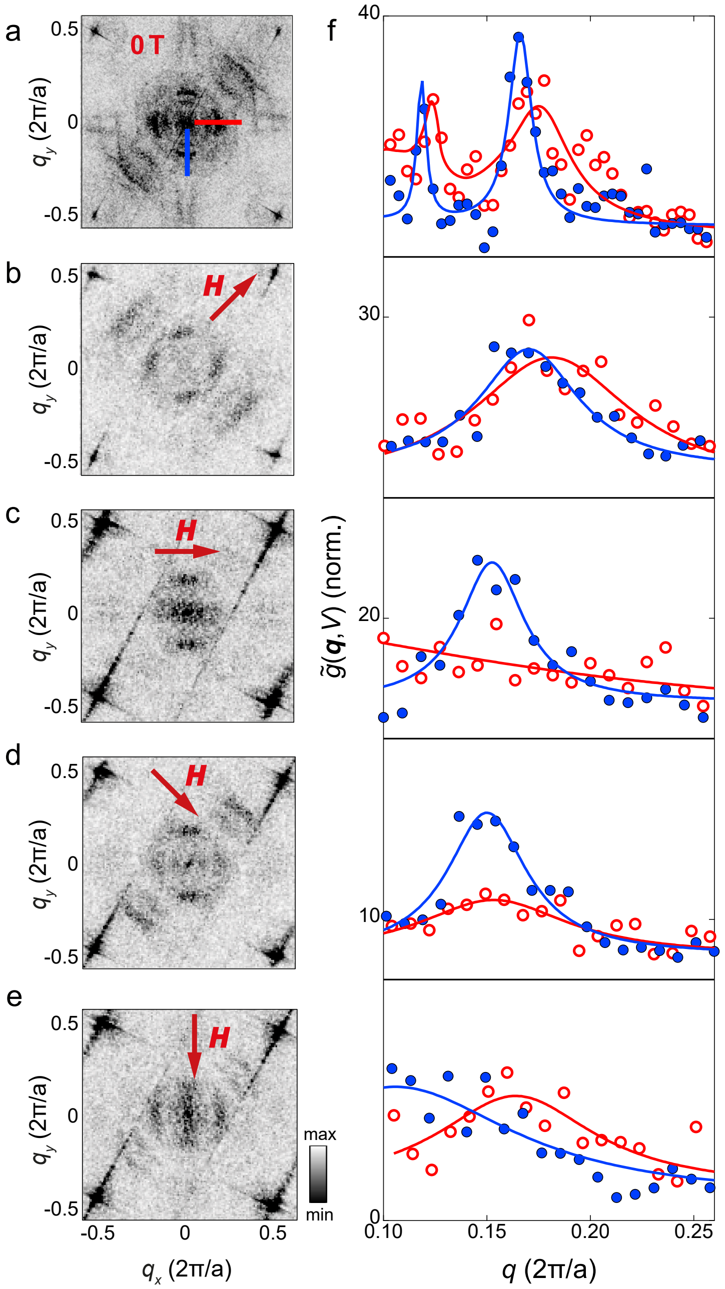

FIG. S6: **Quasiparticle interference imaging. a-e**. Fourier transform of spectroscopic map, shown at **a**. $V = -0.3$mV ($V_\mathrm{s} = 8$mV, $I_\mathrm{s} = 700$pA, $V_\mathrm{L} = 600\ \mu$V, $T = 80$mK, $H = 0$) and **b-e**. $V = 0$mV ($V_\mathrm{s} = 10$mV,$I_\mathrm{s} = 100$pA, $T = 4.2$K, $\mu_0 H = 5$T.) **f**. Line cuts of **a-e**. along $[1\,0\,0]$ (q_x, red) and $[0\,1\,0]$ (q_y, blue) directions. The lines are fitted with Lorentzians as a guide for the eye.

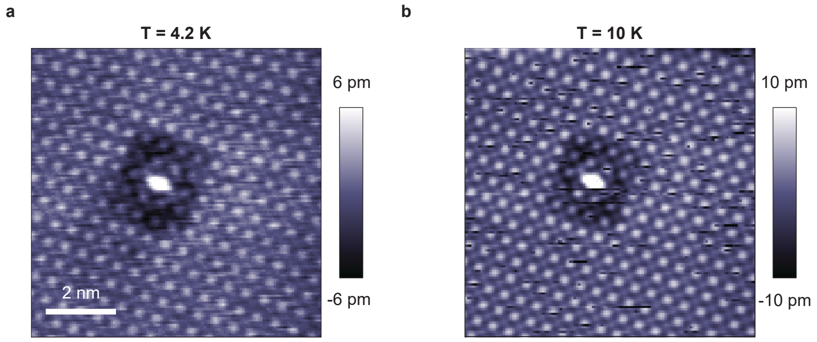

FIG. S7: **Temperature dependence of symmetry breaking a**. Topography of a single defect at $T = 4.2$K. **b**. Topography of the same defect at $T = 10$K.
